# The COVID-19 pandemic as a traumatic experience in the general population

**DOI:** 10.1192/j.eurpsy.2024.1036

**Published:** 2024-08-27

**Authors:** T. Mastelić, V. Višić, T. Borovina Marasović, N. Rančić, D. Vukorepa, M. Milanović, Ž. Kralj, E. Burilović, M. Pernat, K. Glavičić, M. Baković, A. Sabljar, S. Kozina, D. Lasić, M. Mavar, T. Glavina

**Affiliations:** ^1^Psychiatry Department, University Hospital of Split, Split; ^2^Psychiatric Hospital Ugljan, Ugljan; ^3^Psychological medicine, School of Medicine Split, Split, Croatia

## Abstract

**Introduction:**

The COVID-19 pandemic has significantly affected everyday life in most countries of the world. Researches conducted in 2020 showed that COVID-19 was a traumatic experience for 18-60% of respondents in the general population, depending on the country where the research is conducted. Researches for later periods are rare, although we can expect significant changes. We found only one study from 2023 on this topic, although not on the general population.

**Objectives:**

That is why we were interested in what the situation is like after 3 years of the pandemic, when we have been living without non-pharmacological anti-pandemic measures for almost a year.

**Methods:**

The research was conducted at the beginning of 2023. 48 respondents who were not treated psychiatrically or are medical workers were surveyed, because it was shown that these groups were exposed to a greater risk of impaired mental health during the COVID-19 pandemic. To assess the level of traumatic experience, i.e. the risk of developing PTSD as a consequence of the COVID-19 pandemic, we used the Impact of Event Scale With Modifications for COVID-19 (IES-COVID19). A score on that scale of 27 to 34 indicates a clinically significant level of trauma, i.e. there is a 75% chance of developing PTSD. A result of 35 and above suggests that it is necessary to seek professional help.

**Results:**

Our research included 19 (39.6%) men and 29 (60.4%) women. The average age of the respondents is 60.4 years. 29 (60.4%) respondents know that they have recovered from COVID-19. 2 (4.2%) subjects were treated in the hospital due to COVID-19. 8 (16.7%) respondents have a traumatic experience of the COVID-19 pandemic. 5 (10.4%) respondents are in the category of clinically significant level of trauma, while 3 (6.3%) respondents are in the category that should seek professional help. The group traumatized by COVID-19 does not have significantly more respondents who recovered from COVID-19 (p=0.510) nor does it differ in terms of gender representation (p=0.984).

**Conclusions:**

At the beginning of the COVID-19 pandemic, there were discussions about whether it can even be classified as a traumatic experience and whether we can talk about PTSD as a consequence of the pandemic. With this time lag, it seems that in part of the population we are finding PTSD symptoms that are a consequence of the pandemic, but to a lesser extent than research at the beginning of the pandemic suggested. Certainly, additional research is needed on this topic. Also, it is necessary to examine risk factors for possible prevention, as well as therapeutic possibilities.

**Disclosure of Interest:**

None Declared

